# Supplementary material for: Dissociating spatial frequency reliance from adversarial robustness advantages in neurally guided deep convolutional neural networks
Source: ArXiv. 2026 May 6:arXiv:2605.04443v1. Preprint. [Version 1] (PMC13178717)
Supplement: 1 [file NIHPP2605.04443V1-supplement-1.pdf]

Supplemental Information

Fig. S1

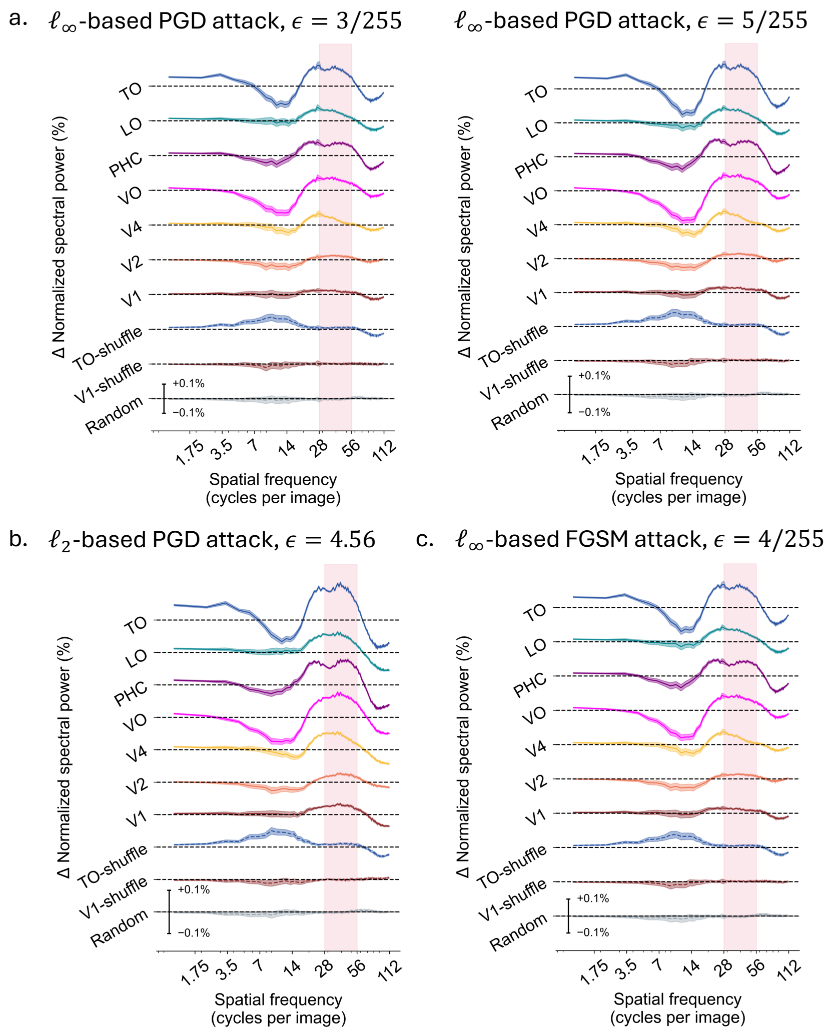

Spatial frequency reliance profiles, shown as differences relative to the baseline model, for NG and control models estimated under alternative adversarial attack parameters. Profiles shown are each computed using a. different perturbation magnitudes ϵ , b. an ℓ_2-bounded attack instead of ℓ_∞, and c. the Fast Gradient Sign Method (FGSM). Across all conditions, the resulting profiles closely match those shown in Fig. 2a (right panel), which were obtained using an ℓ_∞-

bounded PGD attack with $\epsilon = 4/255$. These results indicate that the observed spatial frequency reliance patterns are agnostic to the choice of adversarial attack method and parameterization.

Fig. S2

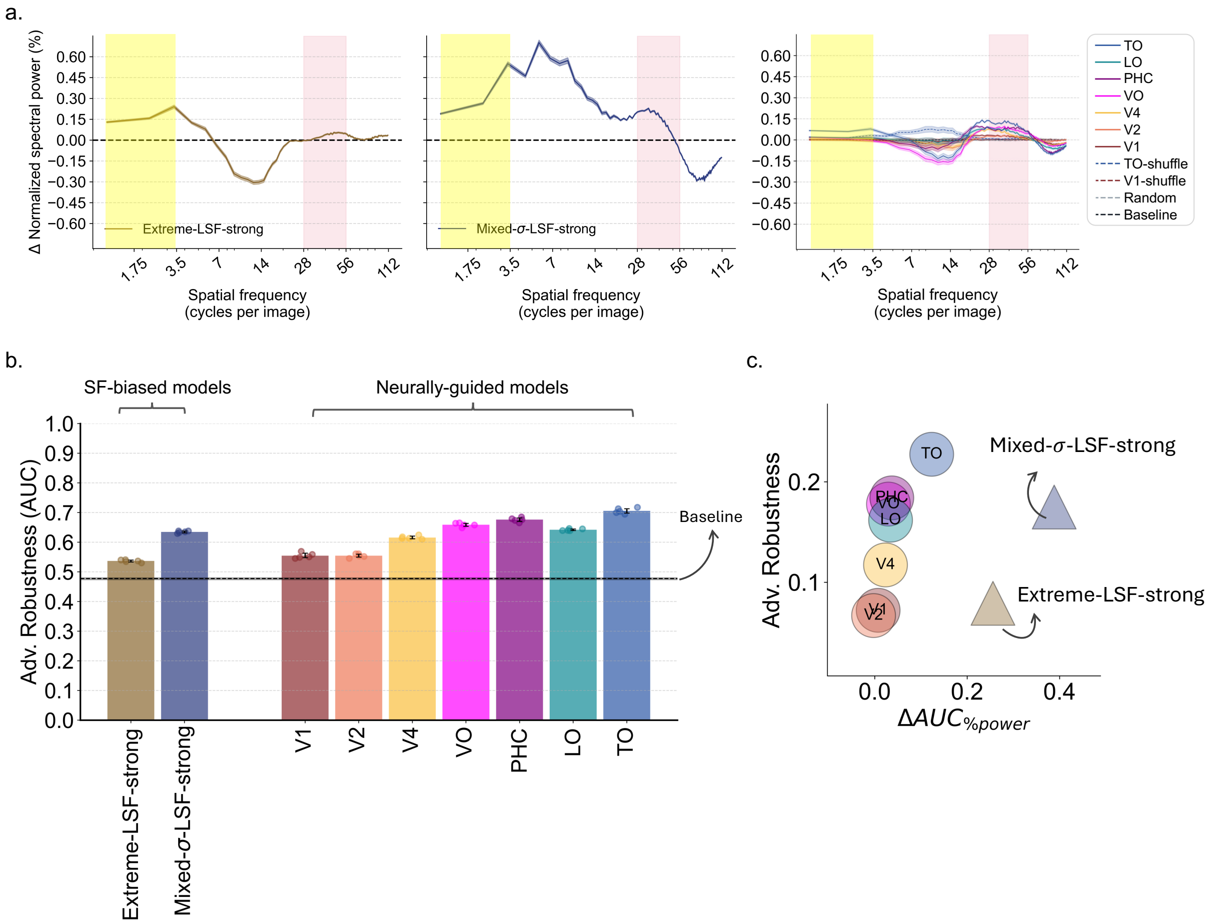

Comparing the magnitude of SF reliance shifts and adversarial robustness gains in LSF-biased and neurally guided models. To directly compare LSF-biased models with the neurally guided (NG) models, we trained a separate set of LSF-biased models maximally matching the training recipe used in the previous study [12]. This matched comparison was necessary because the LSF-biased models analyzed in the main text were trained under different conditions. Most notably, as described in the Methods, all models throughout the current work, except this particular set, are trained from scratch rather than from pretrained weights as the NG models do. We adopted from-scratch training throughout because the SF biases inherited from pretrained

weights were difficult to override, even with full-model fine-tuning. Consistent with this, the LSF-biased models shown here exhibit smaller shifts in SF reliance than the models shown in the main text (Fig. 3d).

Here, we focused on the two LSF-biasing conditions that produced the largest robustness gains in Fig. 3c, namely the strongly biased extreme-LSF model and the strongly biased mixed-σ-LSF model. For the extreme-LSF condition, we increased the proportion of filtered images needed to 80% (as opposed to 50% used in the main text) to induce a sufficiently strong bias, while keeping the filtering parameters otherwise matched to the main-text setting.

a. We first show the SF reliance profiles for the strongly biased extreme-LSF model, the strongly biased mixed-σ-LSF model, and the NG models. The human channel and the LSF range are highlighted in pink and yellow, respectively. Notably, both the LSF-biased models show substantially larger shifts in SF reliance than the NG models. The extreme-LSF model shows approximately a two-fold larger shift, whereas the mixed-sigma LSF model shows shifts of up to four-fold larger.

b. Adversarial robustness, summarized as robust AUC, for the two LSF-biased models and the seven NG models. Dots indicate performance of models trained using five different random seeds, and error bars denote the 95% confidence interval across seeds. Despite their much larger shifts in SF reliance, the LSF-biased models show only modest robustness gains. The extreme-LSF model does not outperform any of the NG models. The mixed-sigma LSF model performs

better, but only reaches the level of the LO-guided model, even though the LO-guided model shows only a minimal shift in the LSF range. This dissociation indicates that increased LSF bias alone cannot account for the robustness advantage conferred by neural guidance.

c. Scatter plot comparing SF reliance shifts (from a.) and adversarial robustness gains (from b.) across NG models and LSF-biased models, similar to that shown in Fig. 2b. The LSF-biased models are shaped as triangles for visibility. The dissociation between SF reliance shifts and robustness gains is readily visible: NG models cluster along the y-axis with relatively small LSF shifts but a wide range of hierarchical robustness gains, whereas the LSF-biased models sit far to the right with substantially larger LSF shifts yet smaller robustness gains.

Fig. S3

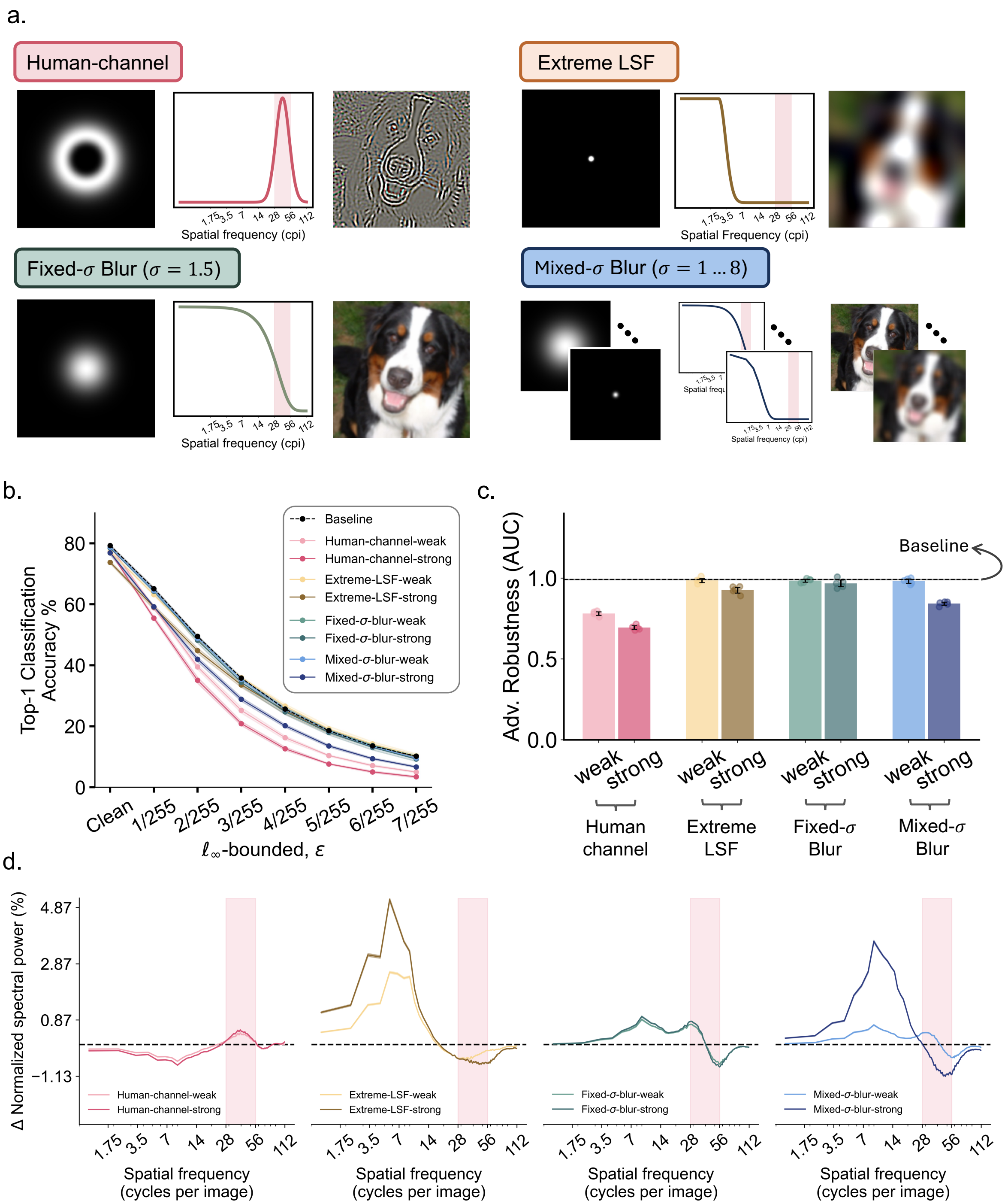

Filtering-based spatial frequency (SF) biasing does not cleanly manipulate SF reliance and does not improve adversarial robustness. Filtering-based methods to manipulate spatial

frequencies in natural images are more commonly used in the field [16,40]. However, we used the phase-scrambling-based method in the main experiments because we found that filtering-based biasing does not produce clean manipulations of SF reliance. We illustrate this here by repeating the same experiments shown in Fig. 3 using filtering-based SF biasing methods.

a. We first show the visualization of the SF filters and corresponding filtered images used to impose filtering-based SF biases during training. Similar to Fig. 3a, for each condition, we show the 2D frequency-domain filter mask (left), the corresponding 1D magnitude response plotted as a function of SF in cycles per image (cpi) on a logarithmic scale (middle), and example filtered images (right). The four conditions are: 1. the human-channel bandpass filter; 2. an extreme low spatial frequency (LSF) low-pass filter preserving frequencies below 3.5 cpi; 3. a fixed-σ blur condition using a Gaussian kernel with standard deviation $\sigma = 1.5$, following [11]; and 4. a mix-σ blur condition implemented as a mixture of Gaussian kernels with different σ values, following [16]. We illustrate two example kernels here, $\sigma = 1.0$ and $\sigma = 8.0$. The human channel is highlighted by the pink shaded region in all 1D magnitude response plots. **b**. Adversarial robustness performance of models trained under each filtering-based SF bias condition. In all conditions except for baseline, we again included weak and strong SF bias variants by varying the proportion of filtered images during training. We used the same proportions (30% for weak bias and 50% for strong bias) as in the phase-scrambled-based experiments. All other training details are also kept identical. Top-1 classification accuracy under ℓ_∞-bounded PGD adversarial attacks [37] is shown across a range of perturbation bounds ϵ. Shaded regions indicate bootstrapped 95% confidence intervals (CI) estimated from five independent models each trained with a different random seed. **c**. Area under the robustness

curve (AUC) summarizing adversarial performance for each condition shown in **b**., with higher AUC indicating greater robustness. Error bars denote bootstrapped 95% CI.

d. SF reliance profiles for all filtering-based SF-biased models, shown as differences relative to the baseline model, similar to Fig. 2a. From left to right, conditions are human-channel-biased, extreme-LSF-biased, fixed-σ-biased, and mix-σ-biased. Positive values indicate greater reliance at a given SF relative to the baseline model. Weak and strong variants within each condition are plotted together. The human channel is highlighted by the pink shaded region.

Our results show that filter-based biasing did not elicit clean, selective SF biases. For example, the human-channel condition showed only a relatively small shift in reliance, whereas the extreme-LSF condition showed its most prominent increase in reliance in additional octaves beyond the targeted range. Furthermore, none of the filtering-based models showed improved adversarial robustness relative to the baseline, which further supports the conclusion that simply biasing models towards selected SF ranges is not sufficient to explain the robustness advantage associated with neural guidance. One possible reason is that filtered images only reduce the availability of untargeted SF cues, that is, SF ranges outside the targeted band, rather than actively discouraging reliance on them. DCNNs are well known to exploit any predictive cues that remain available [57], and thus filtering-based methods may not impose a strong enough bias. By contrast, the phase-scrambling-based method not only preserves the targeted band but also actively disrupts the untargeted bands, thereby imposing a stronger bias. Therefore, we used the phase-scrambling-based SF biasing method in the main experiments.
